# Mediating Processes in the Relations of Parental Monitoring and School Climate With Cyberbullying: The Role of Moral Disengagement

**DOI:** 10.5964/ejop.v15i3.1742

**Published:** 2019-09-27

**Authors:** Maria Giuseppina Bartolo, Anna Lisa Palermiti, Rocco Servidio, Pasquale Musso, Angela Costabile

**Affiliations:** a Department of Cultures, Education and Society, University of Calabria, Arcavacata di Rende, Italy; Trinity College Dublin, Dublin, Ireland

**Keywords:** cyberbullying, moral disengagement, parenting, school climate, mediating processes

## Abstract

In recent years, studies have extensively explored both personal and environmental predictors of cyberbullying. Among these predictors, parental monitoring and school climate were often expected to be associated with cyberbullying behaviors. However, little is known about the mediating mechanisms through which these relations may develop. The present study aimed to expand the current research by testing a theoretical model including the mediating role of moral disengagement in the relations between parental monitoring (including less collaborative vs. more collaborative strategies), school climate, and cyberbullying behaviors. Five hundred and seventy-one Italian adolescents (54.5% male) aged 14 to 20 years were recruited from high schools. Measures included demographics and parental monitoring, school climate, moral disengagement and cyberbullying scales. To test the hypothesized model, we estimated full and partial mediation models by structural equation modeling. Results showed negative indirect links of parental monitoring (but only the more collaborative strategies) and school climate with cyberbullying via moral disengagement. Less collaborative strategies of parental monitoring were neither directly nor indirectly related to cyberbullying. The findings revealed moral disengagement as an important process in explaining how ecological factors, such as parenting behaviors and school environments, are associate with cyberbullying. Limitations, strengths, and implications for practice are presented.

In recent years, cyberbullying has increasingly interested researchers (see Slonje & Smith, 2008, for a seminal paper; Hutson, Kelly, & Militello, 2018) since adolescents use internet with increasing frequency, by their smartphones or other digital devices, not only to establish and maintain their relationships, but also to bully each other. Cyberbullying is defined as a behavior occurring in the online space that willfully and repeatedly intends to inflict harm or discomfort on others by hostile or aggressive messages or acts (Campbell, 2005; Olweus & Limber, 2018), representing a well-recognized critical social concern. This is why most studies have investigated factors to prevent cyberbullying among adolescents and/or tried to identify new forms of interventions. Specifically, research has focused on exploring specific predictors of cyberbullying, both personal and environmental (Chen, Ho, & Lwin, 2017). Among these predictors, parental monitoring and school climate were often expected to be associated with cyberbullying behaviors (see Baldry, Farrington, & Sorrentino, 2015, for a review). However, findings from the literature are controversial.

With regard to parental monitoring, some studies underlined the negative association with cyberbullying (e.g., Baldry et al., 2015), but other studies found a positive association (Álvarez-Garcia, Pèrez, Gonzàlez, & Pérez, 2015; Sasson & Mesch, 2017); however, the literature has also quite consistently shown a negative association between the related dimension of child disclosure and cyberbullying (e.g., Shapka & Law, 2013). An explanation for these inconsistent results might be the broad conceptualization of parental monitoring. Usually, it is thought of as an umbrella term for indicating parental solicitation, control and knowledge, which are flanked by adolescent’s disclosure (Stattin & Kerr, 2000). Potentially, these specific dimensions of parental monitoring may be interpreted in terms of how they are associated in different ways with cyberbullying behaviors, with more collaborative aspects (e.g., parental knowledge and adolescent’s disclosure taken together) more likely to be negatively related to cyberbullying.

As for school climate, although a number of studies found a positive relation between negative school climate and cyberbullying behaviors (see Guo, 2016, for a recent meta-analysis), it is not still clearly known which specific components of school climate are related to cyberbullying. In fact, school climate has been conceptualized and measured in different ways (e.g., unidimensional vs. multidimensional, see Wang & Degol, 2016) and these different views led to observed relations between school climate and cyberbullying that are not easily interpretable.

Taken together, these results call for further study in order to shed light on the relations of parenting behaviors and school climate dimensions with cyberbullying. Yet, little is also known about the mediating mechanisms through which these relations may develop. Particularly, despite the crucial associations linking moral disengagement with parental behaviors (e.g., Meter & Bauman, 2018), school environment (Pozzoli, Gini, & Vieno, 2012), and peer aggression and cyberbullying (e.g., Gini, Pozzoli, & Bussey, 2015; Renati, Berrone, & Zanetti, 2012), no study, to our knowledge, has examined moral disengagement as a mediator among these constructs.

In light of this, the present study aimed to expand the current research in this area by testing a theoretical model including the mediating role of moral disengagement in the relations between parental monitoring, school climate, and cyberbullying behaviors. In doing so, we paid particular attention to how to conceptualize, by using both theoretical and methodological reasons, the proposed constructs, in order to prevent the potential problems already mentioned.

## Cyberbullying Aggression and Victimization

Cyberbullying is a phenomenon characterized by negative social relationships through digital devices and internet (Kowalski, Limber, & Agatston, 2008; Smith et al., 2008). It maintains some specific characteristics of bullying such as intentionality and the imbalance of power (see Smith, 2014; Smith et al., 2008), but it occurs in cyberspace environments so that it reaches a far wider audience at rapid speed (e.g., Kowalski, Morgan, & Limber, 2012). It manifests in different forms such as online harassment, denigration, happy slapping, trickery, impersonation and it is characterized by anonymity, stimulating more disinhibited behaviors and making cyberbullies’ identity masked to their victims (Suler, 2004). Usually, the negative cyberbullying messages gain popularity through social networking sites due to their multitude of users, who can simply witness or actively participate in the process. As a consequence, even when cyberbullies stop their aggressive acts, other different people may continue to spread the harmful contents (e.g., Smith, 2012), creating a dangerous loop that can have serious social and health implications.

Although there is substantial agreement on many of these aspects, the literature on cyberbullying also evidences some important differences. Particularly, there are studies that identify cyberbullying exclusively as cyber-aggression (e.g., Calvete, Orue, Estévez, Villardón, & Padilla, 2010) and other studies as cyber-victimization (e.g., Müller, Pfetsch, & Ittel, 2014). However, when considering the cyberbullying phenomenon, there are not only aggressors or victims as the substantial characters but also the bully-victims, who are aggressors and victims at the same time (see Yang & Salmivalli, 2013). Therefore, there exists a dynamic nature of cyberbullying and related roles (see Del Rey et al., 2015), and studying only specific parts of the phenomenon (cyberbullying aggression or victimization) omits its complexity. Our study took into account this dynamic and conceptualized cyberbullying as including both cyber-aggression and cyber-victimization dimensions that, in turn, imply intersectional behaviors and roles.

## Parental Monitoring: Characteristics and Relations to Cyberbullying

During adolescence, parent-child interactions usually tend to be more conflicted and less warmth (e.g., Branje, 2018). However, the support of parents and positive mutual communication still play a crucial role in the life of adolescents in terms of promoting social competence and preventing problem behaviors (e.g., Padilla-Walker & Son, 2018). Parents often try to be involved in their adolescent children’s life through a variety of monitoring activities. They may control their teens, demanding direct information about or trying to influence their activities or relationships (Stattin & Kerr, 2000). Also, they can solicit information from them or from knowledgeable others, such as friends, neighbors or teachers (Waizenhofer, Buchanan, & Jackson-Newsom, 2004). These two strategies along with one of their potential products, that is parental knowledge of out of home activities, have usually been conceptualized as parental monitoring, defined as “a set of correlated parenting behaviors involving attention to and tracking of the child’s whereabouts, activities, and adaptations” (Dishion & McMahon, 1998, p. 61). However, parental knowledge also derives from adolescents’ spontaneous disclosure about their life activities, relationships, and entertainments; accordingly, research identified adolescent’s disclosure as a strong predictor of how much parents know (Keijsers, Branje, Frijns, Finkenauer, & Meeus, 2010; Stattin & Kerr, 2000).

Starting from this framework, parental monitoring has been studied in association with cyberbullying with findings being discrepant across studies (see, e.g., Álvarez-Garcia et al., 2015). A potential interpretation for these inconsistencies is that it should be taken more into account the nature of strategies used by parents. For example, Elsaesser, Russell, McCauley Ohannessian, and Patton (2017) found that strategies focused on parental control are only weakly related to cyberbullying victimization and perpetration; on the contrary, strategies that are more collaborative (e.g., mediation strategies) are more closely connected to cyberbullying behaviors. Also, Zurcher, Holmgren, Coyne, Barlett, and Yang (2018) showed that authoritarian parenting style (characterized by coercion and hostility) served as a risk factor for cyberbullying engagement, whereas authoritative parenting behaviors (warmth and support, reasoning, and democratic participation) were associated with lower levels of cyberbullying.

In line with these considerations, our point of view is that parental monitoring activities may be also viewed in the context of less collaborative vs. more collaborative strategies. Specifically, parental control and solicitation, as active parent requests, may be considered as less collaborative strategies, while the interplay between parental knowledge and adolescent’s disclosure (the strongest source of parental knowledge in adolescence; see, Stattin & Kerr, 2000) may be considered as a more collaborative strategy. Based on this, we hypothesized that active parent requests were not or only weakly related to cyberbullying and parent-child collaborative knowledge was linked to lower levels of cyberbullying.

## School Climate and Cyberbullying

Cohen, McCabe, Michelli, and Pickeral (2009) affirms that school climate “refers to the quality and character of school life. It is based on patterns of people’s experiences of school life and reflects norms, goals, values, interpersonal relationships, teaching and learning practices, and organizational structures” (p. 182). It may be therefore conceptualized as a multidimensional construct including diverse dimensions of school environment, such as teacher support, structure and clarity of rules and expectations, student commitment to achievement, positive peer interactions, negative peer interactions, disciplinary harshness, instructional innovation, student participation in decision making, support for cultural pluralism, and safety problems (see Brand, Felner, Shim, Seitsinger, & Dumas, 2003). However, although studies appear to support this multifaceted conceptualization of school climate, evident limitations exist (see Wang & Degol, 2016, for a review). As an example, there seems to be little clarity about the number of dimensions characterizing school climate as well as a lack of theoretical or empirical reasoning for their inclusion or exclusion. In any case, many studies focus only on one or two domains in their research and the common practice is to use a single scale or combined averages of different scales for measurement issues. This makes it difficult to see which dimensions, or combinations of dimensions, have the most impact on different forms of student outcomes. This is also the case for cyberbullying.

School climate and its perceptions are theoretically related to cyberbullying. From a socio-ecological approach, cyberbullying may result from a number of personal and contextual influences (Cross et al., 2015) and school represents a significant environment in the lives of youth where individuals relate to each other in different ways, e.g., involving peer relationships, teacher attitudes within the classroom, and rules (Kohl, Recchia, & Steffgen, 2013). For these reasons, school climate has been described as a risk and protective factor for cyberbullying behaviors (e.g. Epstein & Kazmierczak, 2006). However, although research has quite consistently found an inverse relation between positive school climate and cyberbullying (e.g., Hinduja & Patchin, 2012), more studies are needed to clearly determine the specific components of school climate which are related to cyberbullying experiences.

Based on a recent work of Acosta et al. (2018), we conceptualized positive school climate as a multidimensional construct including positive peer relationships, good teacher behaviors, rule fairness and clarity, and school engagement to achievement. These dimensions have been demonstrated to be negatively related to aggressive/violent behaviors and cyberbullying (see, e.g., Betts, 2016; Casas, Del Rey, & Ortega-Ruiz, 2013). Therefore, we hypothesized that higher levels of positive school climate, as defined here, was negatively associated with lower levels of cyberbullying.

## The Mediating Role of Moral Disengagement

Beyond the questions raised above, little is also known about the mediating processes through which both parental monitoring and school climate dimensions are associated with cyberbullying. To date, no research seems to have been specifically conducted to explore this issue, even though recent studies have been interested in better understanding the mechanisms involved in the links between some aspects of parenting and antisocial behaviors (e.g., Bao, Zhang, Lai, Sun, & Wang, 2015) or between school climate and cyberbullying (e.g., Acosta et al., 2018). Yet, determining the roles of mediators in the relations between parental monitoring, school climate, and cyberbullying would be highly relevant to advance our knowledge in the field and provide guidance for interventions.

One potentially relevant mediator worthy of consideration is moral disengagement (Bandura, 1991, 2002; Barkoukis, Lazuras, Ourda, & Tsorbatzoudis, 2016) because of its associations with parenting, school climate, and aggressive behavior such as cyberbullying (e.g., Gini, Pozzoli, & Hymel, 2014; Meter & Bauman, 2018; Pozzoli et al., 2012; for a critical review, see Kowalski, Giumetti, Schroeder, & Lattanner, 2014). Actually, moral disengagement has been one of the most studied variables in relation to cyberbullying (see Chen et al., 2017) and it has been shown to be closely associated with cyberbullying behaviors (e.g. Wang, Lei, Liu, & Hu, 2016). Bandura (1999) conceptualized moral disengagement as a set of cognitive mechanisms (such as moral justification, displacement of responsibility and diffusion of responsibility, and dehumanization of the victim) through which humans sometimes endorse conducts conflicting with moral standards without having a bad conscience. In connection with this definition, research showed that cyberbullies, and also cyberbully-victims (although they are also victims, like “pure” cyberbullies, they are perpetrators; see Renati et al., 2012), may be less inclined to feel remorse or guilt; hence, they may report significantly higher levels of overall moral disengagement than school bullies or nonbully peers (e.g., Pornari & Wood, 2010; Slonje, Smith, & Frisén, 2012). One possible explanation is that the lack of face-to-face contact may reduce social responsibility, thus making it easier for users to engage in cyberbullying behaviors (Wachs, 2012). However, some other studies did not find significant associations between moral disengagement and cyberbullying (e.g., Perren & Gutzwiller-Helfenfinger, 2012), but they are lesser in number. In that case, authors attributed such a finding to the lack of need to use cognitive distancing strategies since the victims are not concretely visible. However, these inconsistent results may be related to measurement issues and require further investigation (Perren & Gutzwiller-Helfenfinger, 2012).

With regard to the links between parenting and moral disengagement, both theory and research support these relations. Social cognitive and interactionist theories propose that proximal social contexts are likely to be important predictors of moral disengagement. Parents represent the main social agents for socialization and, therefore, for social and moral development. Research confirmed this link. For example, positive parenting style (Pelton, Gound, Forehand, & Brody, 2004) and attachment (Bao et al., 2015) were found negatively connected with moral disengagement. Also, positive relations between rejecting or poor parenting and moral disengagement were evidenced (see, Campaert, Nocentini, & Ersilia Menesini, 2017; Hyde, Shaw, & Moilanen, 2010). In terms of our conceptualization of parental monitoring, these findings seem to suggest that more positive and collaborative communication strategies between parents and their children (which we refer to as parent-child collaborative knowledge) may be negatively related to moral disengagement, while less collaborative parental strategies (active parent requests) may be only slightly or not associated with moral disengagement. In fact, it is plausible that in the first case more occasions to discuss daily events are implied, promoting the role of parents as agents of moral socialization (Smetana, 2011). Instead, in the second case these opportunities are limited or missing.

Concerning the link between school climate and moral disengagement, this relation seems to be understudied. Nevertheless, there are different arguments to take it into account. As mentioned in the previous paragraph, moral disengagement is not only an individual (internal) process, but social (external) influences can also play an important role in its development (Bandura, 2002). Beyond the family, school represents another relevant socialization agency, where children and adolescents spend most of their daily time across their developing years. At school, they meet other individuals, like teachers and peers, as well as organizational rules and achievement goals that, taken together and seen as a whole, constitute the community and the culture in which students may cultivate social behaviors and moral reasoning (Pozzoli et al., 2012). This process of social influence can be particularly pervasive (e.g., Bronfenbrenner, 1979). However, it seems to depend on the climate characterizing the school environment, that is on the “interactions among and between adults and students and individuals’ beliefs and attitudes” (Wang, Berry, & Swearer, 2013, p. 297). Accordingly, school climate may affect students’ moral attitudes and behaviors. Following this line of thought, we expected that positive school climate may be negatively related to moral disengagement.

All considered, we hypothesized that moral disengagement may be a relevant mediator between the associations of parenting and school climate with cyberbullying. Specifically, we expected negative relations of parent-child collaborative knowledge and school climate, as well as only weak or no association of active parent requests, with moral disengagement. In turn, we predicted a positive link between moral disengagement and cyberbullying.

## Gender and Age Differences

Gender and age have been largely considered significant predictors or control variables of cyberbullying. However, the literature shows mixed results. Some studies have revealed no statistically significant difference between girls and boys in rates of cyberbullying perpetration or victimization (e.g., Fletcher et al., 2014; Lazuras, Barkoukis, Ourda, & Tsorbatzoudis, 2013; Smith et al., 2008), other research has found males (e.g., Fanti, Demetriou, & Hawa, 2012) or females (e.g., Palermiti, Servidio, Bartolo, & Costabile, 2017; Pornari & Wood, 2010) more likely to cyberbully. As far as age is concerned, research shows that “adolescence is a peak period for involvement in cyberbullying” (Slonje et al., 2012, p. 28) and that older students are more likely to engage in cyberbullying due to the increasing access to online environments (e.g. Gradinger, Yanagida, Strohmeier, & Spiel, 2015; Smith et al., 2008). Nevertheless, a number of studies support the lack of association between age and cyberbullying (e.g., Lazuras et al., 2013; Smith et al., 2008).

More consistent results seem to have been obtained when considering the relations of parental monitoring behaviors with gender and age. In terms of gender, both theory (e.g., socialization models, Holmbeck, Paikoff, & Brooks-Gunn, 1995) and research (e.g., Elsaesser et al., 2017) suggest that gender may play a critical role in how parents and their children influence each other. Specifically, there are evidences that girls tend to be more involved in their family than boys (Vieno, Nation, Pastore, & Santinello, 2009) and, thus, more affected by parental educational style and practices (Moreno-Ruiz, Martínez-Ferrer, & García-Bacete, 2019). With regard to age, the assumption that individuals, during adolescence, experience an increased need of independence from parental monitoring practices was supported by a recent meta-analysis on this topic (see Lionetti et al., 2018), showing a decline in adolescent’s disclosure and parental control, knowledge and solicitation.

Also, prior research suggest that moral disengagement is correlated with adolescents’ gender. In Bandura’s (1999) theoretical framework, gender differences in moral disengagement do not exist in early years of life, but they manifest quite quickly during the subsequent developing years with boys tending to be more morally disengaged than girls. Research confirmed these predicted gender differences in adolescence (see Thornberg & Jungert, 2014). In contrast, there are no particular evidences of relations between moral disengagement and age (e.g., Bandura, 1996; Pelton et al., 2004).

Finally, school climate seems to be associated with students’ age. In a longitudinal study, Wang and Dishion (2012) found adolescents’ positive perceptions of school climate decreasing during school years and related to an increase in behavioral problems. Also, they showed girls reporting more positive perceptions of school climate than boys (see also Way, Reddy, & Rhodes, 2007). However, current research on gender differences in school climate is quite inconsistent, with some studies showing no gender differences (e.g., Kuperminc, Leadbeater, Emmons, & Blatt, 1997).

In light of this picture, we deemed it crucial to include gender and age while examining the relations of parental monitoring and school climate with cyberbullying as well as the moderating role of moral disengagement in these links. Specifically, in line with the literature, we expected effects of adolescents’ gender and age on parental monitoring behaviors, effects of gender (but not age) on moral disengagement, and effects of age on school climate. We took a more explorative approach regarding the relations of gender and age with cyberbullying and of gender on school climate, considering the mixed findings in literature.

## The Current Study

**Figure 1:**
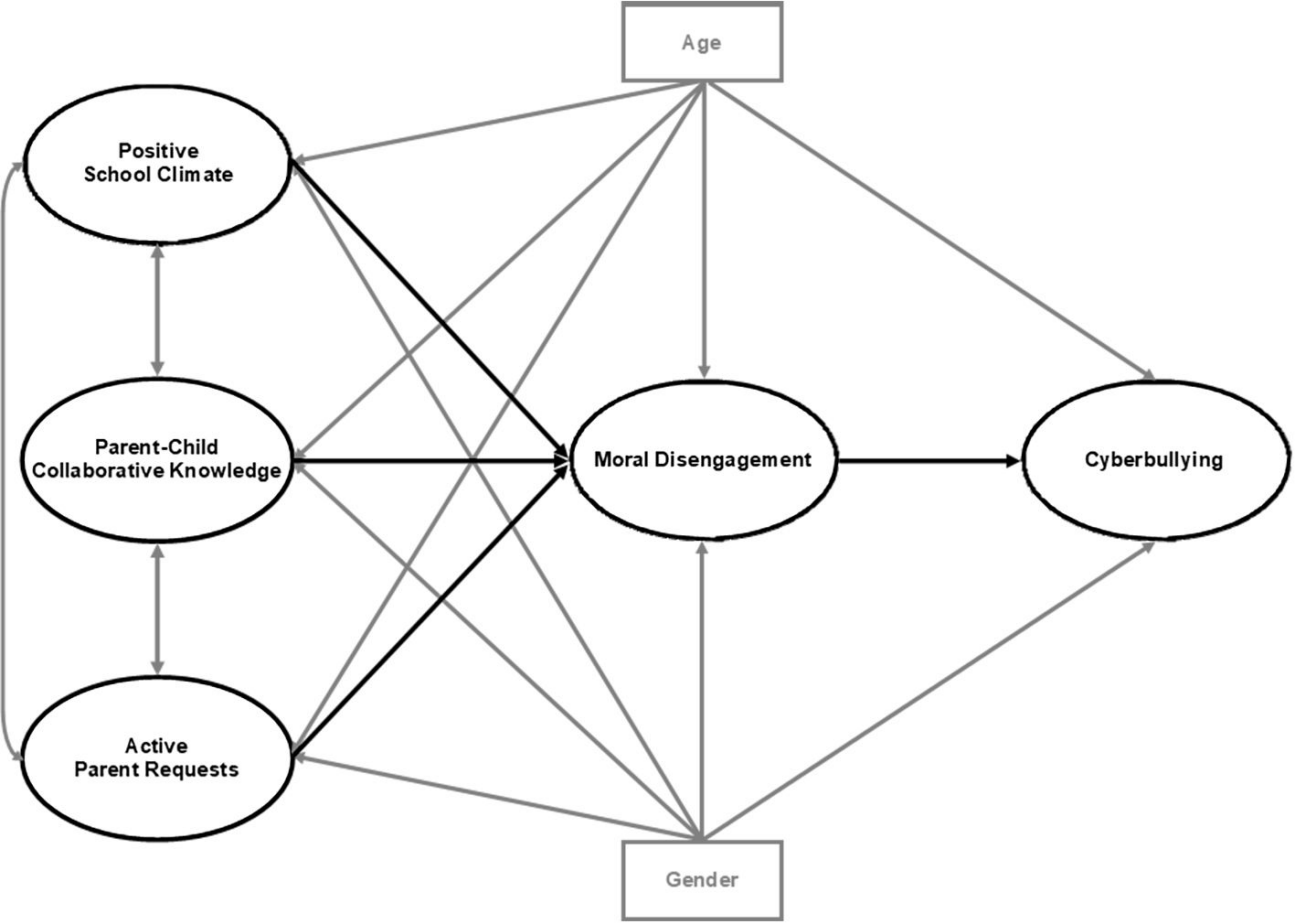
Theoretical mediation model of the relations of parental monitoring behaviors (active parent requests and parent-child collaborative knowledge) and positive school climate with cyberbullying via moral disengagement only. *Note.* The key study variables and their related paths are presented in bold black. Covariances, control variables and their related paths to be included in the analysis are presented in bold gray.

The current study sought to contribute to a more detailed knowledge of the mediating role of moral disengagement between parental monitoring behaviors (including active parent requests and parent-child collaborative knowledge), positive school climate, and cyberbullying. In order to achieve this aim, we tested a theoretical model summarized in Figure 1. As mentioned in the introduction, we hypothesized that:

- active parent requests would be only weakly or not related to moral disengagement;
- parent-child collaborative knowledge would be negatively related to moral disengagement;
- positive school climate would be negatively related to moral disengagement;
- in turn, moral disengagement would be positively related to cyberbullying;
- active parent requests would be only weakly or not indirectly related to cyberbullying via moral disengagement;
- parent-child collaborative knowledge and positive school climate would be indirectly and negatively related to cyberbullying via moral disengagement.

**Figure 2:**
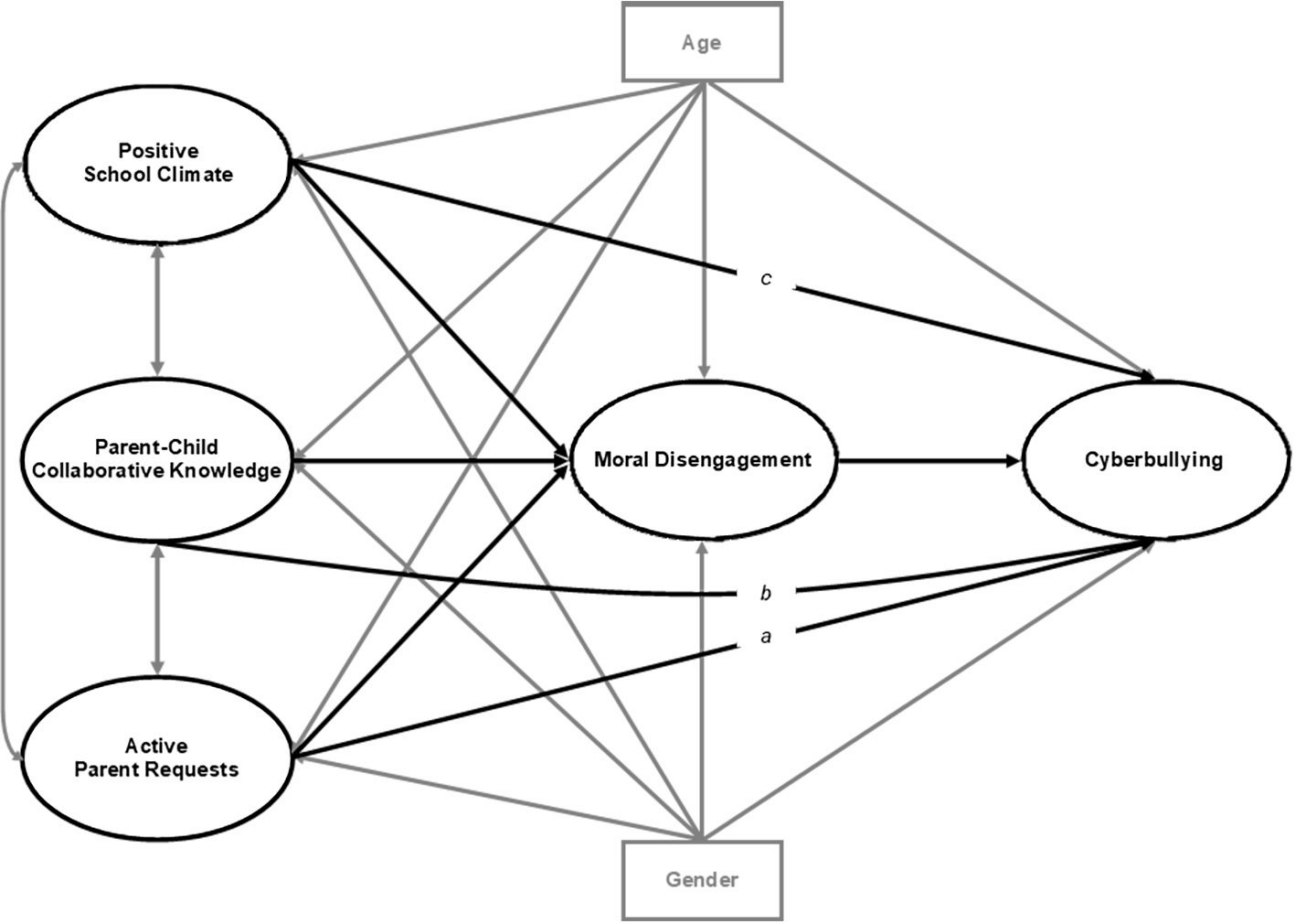
Partial mediation models of the relations of parental monitoring behaviors (active parent requests and parent-child collaborative knowledge) and positive school climate with cyberbullying including both one out of the three direct paths a, b, or c and the indirect paths via moral disengagement. *Note*. The key study variables and their related paths are presented in bold black. Covariances, control variables and their related paths to be included in the analysis are presented in bold gray.

However, in our view, moral disengagement was not necessarily a full mediator of these described relationships. Thus, we also compared this full mediation model with other three partial mediation models, each including a direct link between active parent requests, parent-child collaborative knowledge or positive school climate and cyberbullying (see Figure 2 and the links named a, b, and c, respectively). All models controlled for gender and age, as literature suggested (see previous paragraph).

## Method

### Participants

A sample of adolescents (*N* = 571; males = 54.5% and females = 45.5%) aged 14 to 20 years (*M* = 15.81; *SD* = 1.36) was recruited from five public high schools located in southern Italy (Calabria region). The majority of participants were Italians (94.9%), had cohabiting parents (94.5%), and came from middle-class backgrounds (79.6%) with less than 3% of parents who had elementary school education or lower and less than 18% who had university or post-university education. About 4% experienced parental separation and 1% death of mother or father.

### Procedure

The study and all the procedures were carried out in accordance with the Italian Association of Psychology’s ethical principles for psychological research (2015). All the participants were contacted after randomly selecting 29 classes in the schools willing to participate in the study. The schools were initially selected by an internal university search database, where a list of local school institutions was stored, and encouraged to take part in the investigation through a motivation letter introducing the purpose of the research work. After receiving permission from the respective school-principals, the participants’ parents were informed by letter about the purpose of the research, the voluntary nature of participation and the anonymity of responses. All the parents provided informed consent for their son or daughter’s participation. In addition, participants provided signed assent agreeing to take part in the study. Italian research assistants collected the data after providing a general description of the research aims and measures (particularly, defining what cyberbullying is in terms of aggression and victimization). Participants had about 30 minutes to complete a paper-and-pencil survey during the class time and could withdraw at any time.

### Measures

All the participants completed the validated Italian version of the European Cyberbullying Intervention Project Questionnaire (ECIPQ; Brighi et al., 2012), containing a set of different measures. For the purposes of this study, only some of these measures were used.

#### Socio-Demographics

Respondents were asked to indicate their gender, age, ethnicity, socio-economic status (SES) as well as their parents’ marital status and education.

#### Parental Monitoring

The Parenting Questionnaire (Stattin & Kerr, 2000), as modified from Law, Shapka, and Olson (2010) and adapted in the ECIPQ, was used to assess how parents manage their children's online activities. The original scale contains 16 items asking how often the respondent has experienced particular behaviors related to (a) parental knowledge (four items; e.g., “To what extent do your parents actually know about what you do on the internet and where you are going?”), (b) child disclosure (three items; e.g., “How often do you spontaneously tell your parents about what you are chatting about or posting on the internet?”), (c) parental control (three items; e.g., “To what extent do you have to tell your parents what you will be doing on the internet?”), (d) parental solicitation (three items; e.g., “How often do your parents talk to you about who you are chatting with online?”), (e) parental rule setting (two items; e.g., “Do your parents limit the amount of time you spend on the computer?”), and (f) parental control of internet use (one item; “Do your parents install programs that keep track of where you are going online?”). Except for this last item including a yes/no response set, the other items were scored on a Likert-type scale ranging from 1 (n*ever*) to 5 (*all of the time*). In their study, Law and colleagues (2010) found a two-factor structure including Parent Solicitation and Child Disclosure, after excluding the three items pertaining to rule setting and controlling internet use as they were meant to stand-alone. These factors showed good reliability estimates (Cronbach’s α > .80).

However, because they developed the scale in Canada, it was questionable whether it could be applied to Italian students. Thus, a new latent structure of the items was determined through unweighted least squares exploratory factor analysis using Oblimin rotation. After excluding the only negatively worded item (i.e., “How often do you hide from your parents about what you are doing on the internet?”), analyses suggested two factors explaining 54.88% of the total variance. They were appropriately named as follows: (1) Parent-Child Collaborative Knowledge (PCCK) including six items of parental knowledge and child disclosure and (2) Active Parent Requests (APR) including six items of parental control and parental solicitation. To test the correlated latent factor model of Parent-Child Collaborative Knowledge and Active Parent Requests through robust maximum likelihood confirmatory factor analysis (CFA; see the “Analysis Plan” section for model fit criteria), we used the four composite variables of Parental Knowledge (PK), Child Disclosure (CD), Parental Control (PC), and Parental Solicitation (PS) as observed indicators. After finding good Cronbach’s α values for parental knowledge (.84), child disclosure (.84), parental control (.82), and parental solicitation (.81), we computed the average of their corresponding items, with higher scores indicating higher levels of parental knowledge, child disclosure, parental control, and parental solicitation. Then, we specified Parent-Child Collaborative Knowledge as the latent factor of the observed indicators Parental Knowledge and Child Disclosure as well as Active Parent Requests as the latent factor of the observed indicators Parental Control and Parental Solicitation. This model was well supported, χ^2^_S-B_(1) = 0.15, *p* = .70, CFI = 1.000, RMSEA = 0.000, 90% CI [0.000, 0.081], SRMR = 0.002, and thus used in subsequent analyses.

#### School Climate

The Inventory of School Climate-Student (ISC-S; Brand et al., 2003) was used to assess perceptions of whole school climate. The ISC-S consists of 50 items assessing 10 different dimensions: teacher support, structure and clarity of rules and expectations, student commitment to achievement, negative peer interactions, positive peer interactions, disciplinary harshness, student participation in decision making, instructional innovation, support for cultural pluralism and safety problems. According to our conceptual definition of positive school climate and to limit the number of variables during analyses, in this study we used only four subscales (see Acosta et al., 2018): Teacher Support (TS; six items, e.g., “Teachers go out of their way to help students”), Structure and Clarity of Rules and Expectations (SCRE; five items, e.g., “When teachers make a rule, they mean it”), Student Commitment to Achievement (SCA; five items, e.g., “Students work hard for good grades in classes”), and Positive Peer Interactions (PPI; five items, e.g., “Students get to know each other well in classes”). Items were scored on a Likert-type scale ranging from 1 (*never*) to 5 (*always*). Prior research has provided reliability and validity data for the ISC-S (e.g., Acosta et al., 2018; Brand et al., 2003). In the current study, Cronbach’s α values for teacher support (.74), structure and clarity of rules and expectations (.68), student commitment to achievement (.79), positive peer interactions (.73), and the entire scale (.77) were acceptable. Therefore, for each subscale, a composite variable was created by computing the average of the items, with higher scores indicating higher levels of teacher support, structure and clarity of rules and expectations, student commitment to achievement, and positive peer interactions. These composites were modelled to form the latent factor of Positive School Climate (PSC) to be used in subsequent analyses. This solution was supported through robust maximum likelihood CFA, χ^2^_S-B_(2) = 3.67, *p* = .16, CFI = 0.996, RMSEA = 0.038, 90% CI [0.000, 0.099], SRMR = 0.020.

#### Moral Disengagement

The adolescent version of the Moral Disengagement Scale (MD-S; Bandura, Barbaranelli, Caprara, & Pastorelli, 1996; Caprara, Pastorelli, & Bandura, 1995) was used to assess the degree of acceptance of moral exoneration of detrimental conduct. The scale consists of 24 items assessing six moral disengagement mechanisms: moral justification (“It is alright to fight to protect your friends”), advantageous comparison (e.g., “It is okay to insult a classmate because beating him/her is worse”), displacement of responsibility (e.g., “If kids are living under bad conditions they cannot be blamed for behaving aggressively”), diffusion of responsibility (e.g., “If a group decides together to do something harmful it is unfair to blame any kid in the group for it”), distorting consequences (“It is okay to tell small lies because they don’t really do any harm”), and attribution of blame (“If kids fight and misbehave in school it is their teacher’s fault”). Items were scored on a Likert-type scale ranging from 1 (*strongly disagree*) to 5 (*strongly agree*). Although MD-S measures disengagement of different forms of negative conduct in diverse contexts and interpersonal relationships, prior studies have consistently provided evidence that its items load on a single factor with good internal consistency and have thus used a single score of moral disengagement (e.g., Gini et al., 2015). Following this line, also in this study Cronbach’s α value for the composite measure of moral disengagement was good (.86). Therefore, considering the unidimensionality of the MD-S, we used a parceling approach (Little, Cunningham, Shahar, & Widaman, 2002) for latent modeling. Specifically, items were parceled into four indicators comprising six items, with an equal distribution of factor loadings across parcels. Each parcel was computed by averaging the responses across the six selected items, with higher scores meaning higher levels of proneness to morally disengage. A robust maximum likelihood CFA supported the one-factor structure, χ^2^_S-B_(2) = 4.00, *p* = .13, CFI = 0.996, RMSEA = 0.042, 90% CI [0.000, 0.102], SRMR = 0.013. Hence, the four parcels were modelled to form the latent factor of Moral Disengagement (MD) also in the subsequent analyses.

#### Cyberbullying Aggression and Victimization

The self-report ECIPQ Cyberbullying Scale (ECIPQ-CS; Del Rey et al., 2015) was used to assess two dimensions of cyberbullying behaviors—aggression and victimization—asking how often the respondent had experienced the listed behaviors as perpetrator (11 items; Cyber-Aggression subscale) and victim (11 items; Cyber-Victimization subscale) during the previous 2 months either online or through cell phones. Each subscale covers behaviors such as uploading and/or altering of embarrassing images or video (i.e., “I altered pictures or videos of another person that had been posted online” for aggression), identity theft (i.e., “Someone created a fake account, pretending to be me” for victimization), and indirect abuse (e.g., “Someone spread rumors about me on the internet” for victimization). Items were scored on a Likert-type scale ranging from 1 (*no*) to 5 (*yes, more than once a week*). Reliability and validity data have been provided for the ECIPQ-CS subscales when used with students in cross-cultural studies (e.g., Del Rey et al., 2015). Previous research also supported the use of the ECIPQ-CS as second order measure to obtain a latent variable of Cyberbullying (CB) including Cyber-Aggression (CA) and Cyber-Victimization (CV) as observed indicators (see, e.g., Casas et al., 2013). In the current study, this second-order factor structure was supported through robust maximum likelihood CFA, by specifying Cyberbullying as the second-order factor and Cyber-Aggression and Cyber-Victimization as the first-order factors with the 11 corresponding items loaded on each of them, χ^2^_S-B_(199) = 228.49, *p* = .07, CFI = 0.956, RMSEA = 0.016, 90% CI [0.000, 0.025], SRMR = 0.052. Cronbach’s α values for cyber-aggression (.93) and cyber-victimization (.86) were consistent with prior researches. Therefore, for each subscale, a composite variable was created by computing the average of the items, with higher scores indicating higher levels of cyber-aggression and cyber-victimization. These composites were modelled to form the latent factor of Cyberbullying to be used in subsequent analyses.

### Analysis Plan

Descriptive statistics for the observed variables were obtained using SPSS (Version 23). Specifically, frequencies of each of the cyber-aggression and cyber-victimization response options as well as mean scores, standard deviations and normality statistics of the study’s observed variables were computed. Then, a series of structural equation models were estimated in *Mplus 7.2* (Muthén & Muthén, 2014). First, to obtain bivariate correlations we performed a structural equation model including control (age and gender) and specifying all latent (Parent-Child Collaborative Knowledge, Active Parent Requests, Positive School Climate, Moral Disengagement, and Cyberbullying) variables, as well as all covariances between them. Second, to test the hypothesized model we estimated a full mediation model that specified parent-child collaborative knowledge, active parent requests, and positive school climate as predictors of moral disengagement and then this mediator as predictor of cyberbullying. Third, we estimated three partial mediation models. The first included a direct path from parent-child collaborative knowledge to cyberbullying, the second from active parent requests to cyberbullying, and the third from positive school climate to cyberbullying. These models were to assess whether our proposed moral disengagement mediator fully accounted for relations of parent-child collaborative knowledge, active parent requests, and positive school climate with cyberbullying. All indirect paths through moral disengagement were tested and each model was controlled for age and gender. Following Faraci and Musso (2013), and Kline (2010), multiple indices were used to evaluate model fit (adopted cutoffs in parentheses): the chi-square (χ^2^) test value with the associated *p* value (*p* > .05), comparative fit index (CFI ≥ 0.95), root-mean-squared error of approximation (RMSEA ≤ 0.06) and its 90% confidence interval (CI ≤ 0.05 for the lower bound and ≤ .08 for the upper bound), and standardized root mean square residual (SRMR < 0.08). However, by acknowledging the potential limitation of the χ^2^ test because of its sensitivity to reject the null hypothesis with large sample sizes and complex models (Sass, 2011), we mostly relied on the goodness-of-fit indices. Nested model comparison (the more restrictive vs. the less restrictive models) was used to examine whether or not direct paths of parent-child collaborative knowledge, active parent requests, and positive school climate with cyberbullying were tenable. In this case, the criteria for ascertaining significant differences included significant χ^2^ difference (Δχ^2^ with *p* < .05).

## Results

### Preliminary Analyses

Tables 1 and 2 summarize the descriptive statistics. Table 1 suggests how our sample quite closely reflected the distribution of the cyberbullying phenomenon in the adolescent population (e.g., Del Rey et al., 2016; Italian National Institute of Statistics, 2015). Table 2 shows how cyber-aggression and cyber-victimization were not normally distributed with skewness and kurtosis values >|1.00| (Kline, 2010). As multivariate non-normality was also evidenced (normalized Mardia’s coefficient was 40.96, *p* < .001), the data were subsequently analyzed using the maximum likelihood robust estimation method, including Satorra-Bentler χ^2^ test statistic (χ^2^_S-B_), robust CFI, and robust RMSEA (Satorra & Bentler, 1994).

**Table 1:** Frequencies of the Cyber-Aggression and Cyber-Victimization Response Options

Response Options	Cyber-Aggression	Cyber-Victimization
1. No	87.6%	85.7%
2. Yes, one or two times	8.1%	11.1%
3. Yes, once or twice a month	1.6%	1.6%
4. Yes, about once a week	0.6%	0.6%
5. Yes, more than once a week	2.0%	1.0%

*Note. N* = 571.

**Table 2:** Mean Scores, Standard Deviations, Skewness, and Kurtosis of the Study’s Observed Variables

	*M*	*SD*	Skewness	Kurtosis
1. PK	3.15	1.09	-0.28	-0.75
2. CD	2.47	1.17	0.40	-0.94
3. PC	2.23	1.05	0.57	-0.49
4. PS	2.43	1.04	0.40	-0.56
5. TS	3.20	0.76	-0.21	-0.27
6. SCRE	3.65	0.73	-0.53	0.32
7. SCA	3.39	0.80	-0.27	0.02
8. PPI	3.72	0.74	-0.47	0.04
9. MD_P1	2.66	0.72	0.21	0.29
10. MD_P2	2.46	0.75	0.27	-0.07
11. MD_P3	2.35	0.81	0.39	-0.03
12. MD_P4	2.28	0.78	0.46	0.24
13. CA	1.20	0.52	4.41	22.12
14. CV	1.20	0.36	3.82	21.18
15. Age	15.81	1.36	0.19	-0.82
16. Gender (0 = male; 1 = female)	0.46	0.50	0.18	-1.97

*Note. N* = 571. PK = parental knowledge; CD = child disclosure; PC = parental control; PS = parental solicitation; TS = teacher support; SCRE = structure and clarity of rules and expectations; SCA = student commitment to achievement; PPI = positive peer interactions; MD_P1 = moral disengagement—Parcel 1; MD_P2 = moral disengagement—Parcel 2; MD_P3 = moral disengagement—Parcel 3; MD_P4 = moral disengagement—Parcel 4; CA = cyber-aggression; CV = cyber-victimization.

Bivariate correlations among latent and control variables after estimating the structural equation model specifying all covariances between them are reported in Table 3. The model fit the data well χ^2^_S-B_(85) = 150.59, *p* = .00002, CFI = 0.973, RMSEA = 0.037, 90% CI [0.027, 0.046], SRMR = 0.032. Results showed the following significant associations:

- Parent-child collaborative knowledge was positively correlated with active parent requests and positive school climate and negatively correlated with moral disengagement and cyberbullying.
- Active parent requests were positively correlated with positive school climate, negatively correlated with moral disengagement (no significant association with cyberbullying).
- Positive school climate was negatively correlated moral disengagement and cyberbullying.
- Moral disengagement was positively correlated with cyberbullying.
- Age was negatively correlated with active parent requests and positive school climate (no significant associations with parent-child collaborative knowledge, moral disengagement, cyberbullying, and gender).
- Gender (0 = male; 1 = female) was positively correlated with parent-child collaborative knowledge and active parent requests and negatively correlated with moral disengagement and cyberbullying (no significant association with positive school climate).

**Table 3:** Bivariate Correlations Among Latent and Control Variables of Study After Estimating a Structural Equation Model Specifying All Covariances Between Them

Variable	1	2	3	4	5	6	7
1. PCCK	–						
2. APR	.79***	–					
3. PSC	.19***	.18***	–				
4. MD	-.25***	-.14*	-.32***	–			
5. CB	-.13*	.03	-.34***	.34***	–		
6. Age	.04	-.14***	-.22***	-.07	.08	–	
7. Gender (0 = male; 1 = female)	.35***	.36***	.08	-.18***	-.15***	-.03	–

*Note.* *N* = 571. PCCK = parent-child collaborative knowledge; APR = active parent requests; PSC = positive school climate; MD = moral disengagement; CB = cyberbullying.

*p < .05. ***p < .001.

### Mediation Model

**Figure 3:**
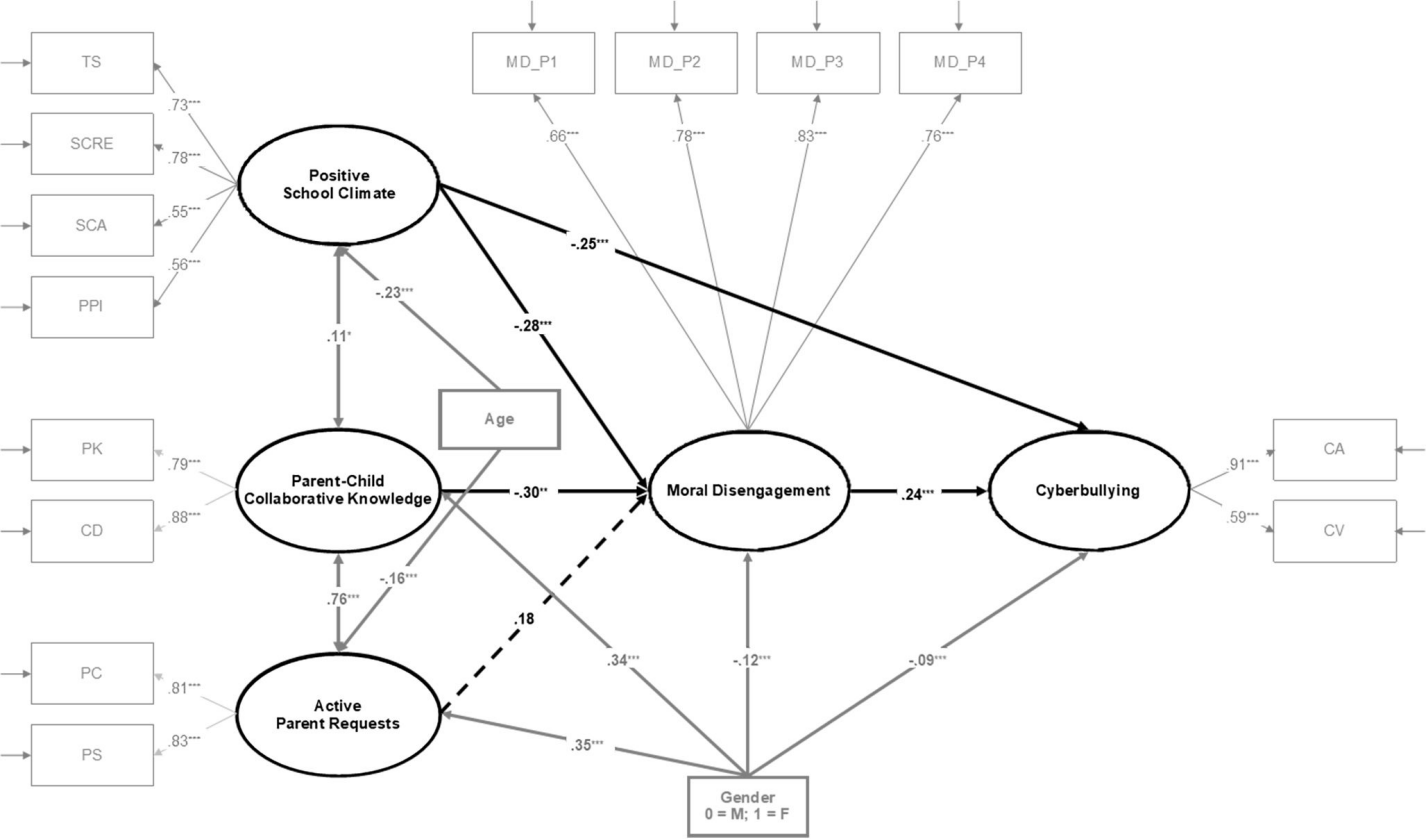
Estimated structural equation model for the best fitting mediation model among the key study variables. *Note.* *N* = 571. Standardized regression coefficients (betas) are shown. PK = parental knowledge; CD = child disclosure; PC = parental control; PS = parental solicitation; TS = teacher support; SCRE = structure and clarity of rules and expectations; SCA = student commitment to achievement; PPI = positive peer interactions; MD_P1, = moral disengagement—Parcel 1; MD_P2 = moral disengagement—Parcel 2; MD_P3 = moral disengagement—Parcel 3; MD_P4 = moral disengagement—Parcel 4; CA = cyber-aggression; CV = cyber-victimization. The measurement part of the model (including observed indicators and factor loadings) is represented in gray. The key study latent variables and their related paths are presented in bold black. Covariances, control variables and their related paths are presented in bold gray. Solid lines represent significant paths and dashed lines represent nonsignificant paths. For better visualization, residuals of latent variables are not shown. The standardized indirect effects of parent-child collaborative knowledge and positive school climate on cyberbullying through moral disengagement were β = -.07^*^ and β = .07^***^, respectively. ^*^p < .05. ^**^p < .01. ^***^p < .001.

We estimated the full and partial mediation models. In the initial estimate of the full mediation model, we controlled for age and gender by allowing them to predict all the latent variables; however, age was only significantly linked to active parent requests and positive school climate, and gender to parent-child collaborative knowledge, active parent requests, moral disengagement, and cyberbullying. Only the significant paths were retained, and the model was re-estimated. This full mediation model fit the data well, χ^2^_S-B_(94) = 187.75, *p* = .00000003, CFI = 0.962, RMSEA = 0.042, 90% CI [0.033, 0.050], SRMR = 0.050. Next, we similarly estimated the three partial mediation models. When comparing the full mediation and the first two partial mediation models including the direct path from active parent requests to cyberbullying and from parent-child collaborative knowledge to cyberbullying, no significant differences were found, Δχ^2^_S-B_(1) = 1.26, *p* = .26, and Δχ^2^_S-B_(1) = 0.04, *p* = .83, respectively. The comparison of the full and the third partial mediation model including the direct path from positive school climate to cyberbullying showed the full mediation model to have a significantly worse fit to the data, Δχ^2^_S-B_(1) = 9.31, *p* = .002. Thus, the third partial mediation model was considered the final model (see Figure 3).

This model showed how parent-child collaborative knowledge and positive school climate were significantly and negatively predictive of the mediator moral disengagement, whereas active parent requests was not significantly associated with moral disengagement. Additionally, positive school climate had a significant and negative direct link to cyberbullying. The mediator was significantly and positively linked to the outcome of cyberbullying. Furthermore, the indirect effects of parent-child collaborative knowledge (β = -.07, *p* = .02) and positive school climate (β = -.07, *p* = .001) on cyberbullying through moral disengagement were significant and negative. No significant direct and indirect associations of active parent requests with cyberbullying were found. Finally, older adolescents perceived lower levels of active parent requests and positive school climate as well as reported higher levels of cyberbullying through the indirect effect of positive school climate (β = .05, *p* = .00008) and of positive school climate and moral disengagement (β = .02, *p* = .005) than younger ones, while female adolescents were more likely to perceive higher levels of parent-child collaborative knowledge and active parent requests and to report lower levels of moral disengagement as well as of cyberbullying both directly and through the indirect effect of moral disengagement (β = -.03, *p* = .04) and of parent-child collaborative knowledge and moral disengagement (β = -.02, *p* = .03) than male ones.

## Discussion

This study aimed to examine the mediating role of moral disengagement in the relations of parental monitoring and school climate with cyberbullying. Theoretical and methodological reasons suggested to conceptualize (a) cyberbullying as a dynamic phenomenon including both cyber-aggression and cyber-victimization (Smith et al., 2008), (b) parental monitoring as including both a more positive and collaborative communication strategy between parents and their children (parent-child collaborative knowledge) and a less collaborative parental strategy (active parent requests) (Stattin & Kerr, 2000), (c) positive school climate as including positive peer relationships, good teacher behaviors, rule fairness and clarity, and school engagement to achievement (Acosta et al., 2018), and (d) moral disengagement as a unidimensional construct (Bandura, 1999). Then, we tested a theoretical mediation model including the indirect paths of active parent requests, parent-child collaborative knowledge, and positive school climate to cyberbullying via moral disengagement. Finally, we compared this full mediation model with three partial mediation models each including a direct path from active parent requests, parent-child collaborative knowledge, or positive school climate to cyberbullying. Findings supported our hypotheses and revealed moral disengagement to be a significant intermediary mechanism for the relations of parent-child collaborative knowledge and positive school climate with cyberbullying. However, while moral disengagement fully mediated the relations between parent-child collaborative knowledge and cyberbullying, a direct link between positive school climate and cyberbullying was also evidenced.

Generally, in terms of definitional and measurement issues, the results of this study sustained the following ideas. First, cyberbullying can be viewed as dynamic in nature, including simultaneously cyber-aggression and cyber-victimization elements (Del Rey et al., 2015; Kowalski et al., 2014). This overlap seems to be in line with the “role inversion hypothesis” (Baldry et al., 2015, p. 44), according to which being a cyber-aggressor is also associated with being a cyber-victim. Second, a conceptualization of parental monitoring in the context of less collaborative vs. more collaborative strategies can be meaningful in order to better distinguish which specific strategies of parental monitoring are associated with lower levels of cyberbullying behaviors. This reflects other studies (e.g., Elsaesser et al., 2017; Zurcher et al., 2018) where it has been suggested that parental strategies focused on authoritarian style or control can be only weakly related to, or even serve as a risk factor for, cyberbullying engagement. In contrast, authoritative style and collaborative strategies are more closely and negatively connected to cyberbullying. Third, it is possible, not only theoretically but also methodologically and practically, to think of perceived school climate as a multidimensional construct (Wang & Degol, 2016), and thus to help to clarify, in line with recent works (e.g., Acosta et al., 2018), which combinations of school climate dimensions can mostly have a negative impact on cyberbullying. Fourth, as reported by Bandura and colleagues (1996), it is reasonable to continue to use moral disengagement as a unidimensional construct.

Focusing on our hypotheses, we predicted that parent-child collaborative knowledge would be negatively associated with moral disengagement (directly) and cyberbullying (indirectly via moral disengagement). On the contrary, we expected that active parent requests would be only weakly or not related to the other two variables. Findings supported our hypotheses. This may provide useful information about why, to date, literature showed discrepant results when investigating the relations between parental monitoring and cyberbullying (e.g., Álvarez-Garcia et al., 2015; Law et al., 2010), with studies reporting parental monitoring as a risk factor (e.g., Meter & Bauman, 2018), and other studies as a protective factor for cyberbullying involvement. In fact, a potential explanation for these inconsistences is related to the differential strategies involved in the parent-child relationship (e.g., Charalampousa et al., 2018; Liga et al., 2017). Close and positive communication interactions between parents and their children, characterized by adolescents’ disclosure and related parental knowledge, appear to be the most effective strategy in preventing involvement in cyberbullying. Conversely, more constrictive parental practices, such as parental control and solicitation, seem not to be effective (or even detrimental under certain circumstances) in making adolescents less susceptible to aggressive behaviors. Therefore, although active parent requests can increase parental knowledge about their adolescent children’s online activities, establishing a positive relational context in which adolescents feel comfortable to share information seems to be a more relevant aspect in decreasing cyberbullying.

This is in line with both the specific and the broader literature. For example, in the specific case, Buelga, Martínez-Ferrer, and Cava (2017) recently found that cyberbullies-victims have low family climate (e.g., conflict) and communication patterns (e.g., non-open and avoidant communication). In the broader case, studies showed that parent-child communication has a great influence on adolescents’ well-being and life satisfaction (Boniel-Nissim et al., 2015). Specifically, an open and honest communication style with parents can facilitate adolescents’ healthy development and prevent antisocial behaviors (Davidson & Cardemil, 2009); instead, an absence of positive parenting is seen as associated with adolescent maladaptive behaviors. However, these considerations need to be thought in light of the mediating role of moral disengagement.

Theories such as social cognitivism and interactionism argue that children’s moral development depends not only on cognitive advancement, but also on the social context that they live in (Smetana, Campione-Barr, & Metzger, 2006). Particularly, parents represent the primary agents of socialization (Nickerson, Mele, & Princiotta, 2008), also in terms of moral values and responsibility, and their parenting style is supposed to influence diverse aspects of their children’s behaviors and thinking (Pelton et al., 2004). Our findings seem to suggest that when adolescents experience a more positive communication environment, able to stimulate self-disclosure with parents and an enrichment of parental knowledge about their online activities, this may result in lower levels of moral disengagement. In such an environment, the occasion of discussing personal or extra-personal daily events, and its moral implications, with parents could be more frequent, implying the cultivation of a more profound moral thinking. In contrast, when the parent-adolescent relationship is characterized by a unidirectional communication exclusively depending on the direct and indirect parents’ requests, this may not impact on moral reasoning formation. This is quite in line with the literature, indicating that diverse forms of positive or negative parenting are differently related to moral disengagement (Bao et al., 2015; Campaert et al., 2017; Hyde et al., 2010). Furthermore, in this study, higher levels of moral disengagement were associated with higher levels of cyberbullying involvement. Hence, it seems that a more limited capability of experiencing remorse and feeling guilt may affect the tendency to aggressive behaviors. This evidence parallels the studies claiming that cyberbullying may be particularly influenced by moral disengagement due to both the lack of face-to-face contact and, therefore, the reduction of social responsibility (Pornari & Wood, 2010; Renati et al., 2012; Slonje et al., 2012; Wachs, 2012). From all these results and related considerations, it is plausible to assume that there may exist an indirect path from parenting behaviors to cyberbullying involvement through the action of moral disengagement. When adolescents are experiencing a positive communication environment with parents, this can produce more opportunities for moral socialization that, in turn, can prevent cyberbullying.

Our expectations also implied that school climate would be negatively associated with moral disengagement (directly) and cyberbullying (indirectly via moral disengagement). Findings supported this prediction, but additionally revealed a direct link between school climate and cyberbullying. Thus, it can be reasonable to think that, when school environment is characterized by a perceived supportive relational atmosphere among peer students, clear and consistent rules with teacher support, and active involvement of the students in achieving school success, it may result in a general climate helping to reduce cyberbullying involvement, both directly and through its relation to moral sensitivity. This is in line with social learning theory (Bandura, 1986) positing that the external environment and the observation of others largely influence individuals’ learning. Hence, the reduction of aggressive behaviors (like cyberbullying) can result from repeated exposure to positive relationship models, support from teachers, clarity of rules and expectations, and prevailing achievement orientation among students at school (Cross et al., 2015). Furthermore, this exposure can lead to changes in cognitive sensitization to attacking others, resulting in more negative moral evaluations of aggressive behaviors (e.g., cyberbullying others), less justification for behavior inconsistent with moral standards and norms, and consequently less moral disengagement and, in turn, less cyberbullying. This interpretation is supported by empirical studies showing that positive school climate can be a protective factor for cyberbullying involvement (e.g., Hinduja & Patchin, 2012) as well as it is related to moral learning (Pozzoli et al., 2012).

Finally, the evidence that gender could specifically influence the key variables of this study contributed to enrich the overarching understanding of the processes involved in explaining cyberbullying involvement. As expected, gender was associated with parental monitoring behaviors and moral disengagement. It was also related to cyberbullying. Female adolescents seem to be more affected by parental practices, less morally disengaged, and less involved in cyberbullying. These results support recent research (e.g., De Caroli & Sagone, 2014; Fanti et al., 2012; Moreno-Ruiz et al., 2019) and, in addition, suggest that gender differences in cyberbullying may be also mediated by other intervening variables, such as parent-child collaborative knowledge and moral disengagement. Similarly, in line with the literature (Lionetti et al., 2018; Wang & Dishion, 2012), our predictions about the links between age and both parental monitoring behaviors and school climate were supported. However, age was significantly associated only with active parent requests (the less collaborative parental strategy), but not with parent-child collaborative knowledge, producing no significant relations between age and cyberbullying through parental monitoring strategies. On the contrary, age was positively and indirectly linked to cyberbullying through positive school climate. Older adolescents perceived less positive school climate, and, in turn, this was associated with higher levels of cyberbullying.

Together, our findings suggest that adolescents that live in a positive family and school ecology, where they can develop an authentic adherence to moral norms and standards, are less susceptible to cyberbullying. As the evidence indicated, positive parent-child relationships (more for girls than boys) and school climate (more for younger than older adolescents) are key factors in this process by maintaining an open communication climate in family as well as appropriate levels of student support, clarity of rules, positive relationships and specific achievement goals at school.

### Limitations, Strengths, and Implications

This study should be considered in light of several limitations. First, the cross-sectional nature of the study design precludes us from clearly concluding the direction of the associations among the study variables. Thus, it would be important to conduct future longitudinal studies in order to draw clearer conclusions about causality and the developmental processes involved. Relatedly, only adolescents aged 14–20 were assessed. Although adolescence is the peaking period for involvement in cyberbullying, it would be extremely interesting to follow the same participants from late childhood to late adolescence to better understand the level of significance of our findings. Second, while we conceptualized and assessed parental monitoring in terms of less collaborative vs. more collaborative strategies, the measure we used was not specifically developed for this intent. We, therefore, encourage future research to replicate this study using more fitting measures. Third, we limited our examination to cyberbullying as an outcome (and not face-to-face bullying) since the parental monitoring measure specifically assessed how parents manage their adolescent children’s online activities. To further deepen our understanding of the bullying phenomenon in its many facets, future research should also incorporate school bullying, by using congruent measures of parental monitoring. Fourth, we collected data only by self-report questionnaires from adolescents, which might have increased bias in our results. A multi-informant approach would be desirable for future investigations.

Despite these limitations, our study contributes meaningfully to the literature because it extends the understanding of the relations between adolescent ecological environments and online aggressive behavior. Specifically, our examination of moral disengagement as a mediating variable advances the literature by allowing us to identify and explicate mechanisms or processes that underlie the relationships of parental monitoring and school climate with cyberbullying. Additionally, we were able to reveal some gender- and age-related associations in these processes. These findings enhance the knowledge of factors that can contribute to the prevention of cyberbullying in adolescence through a perspective that integrates contributions from social cognitive and social learning theories (Bandura, 1986), interactionist theory (Thornberry, 2018), socialization models (Holmbeck et al., 1995), and ecological models (Bronfenbrenner, 1979).

Moreover, findings from this study provide implications for practice, suggesting some guidelines for designing socio-educational programs aimed at reducing cyberbullying involvement. Such programs should have the intention of involving diverse actors at different levels, by providing adolescents, their parents, and their schools with opportunities of more collaborative and ethical environments. Actions should mainly promote improvements in moral development of adolescents, relationship and communication skills of parents, positive educational environments of schools including mutual trust, support, active involvement of students, teachers and school staff. Also, these actions should duly take into account gender and age differences, for example by increasing activities that favor a positive school climate among older adolescents. In the light of this study, interventions intended for one of these actors or considering some aspects of the processes linked to cyberbullying could have some success. However, only a more general and complex intervention program, able to simultaneously take into consideration the various ecological life contexts of the adolescent, seem to be able to ensure better possibilities to effectively prevent a concerning and ever-expanding social phenomenon such as cyberbullying.
